# Disease diagnostic method based on cascade backbone network for apple leaf disease classification

**DOI:** 10.3389/fpls.2022.994227

**Published:** 2022-09-23

**Authors:** Xing Sheng, Fengyun Wang, Huaijun Ruan, Yangyang Fan, Jiye Zheng, Yangyang Zhang, Chen Lyu

**Affiliations:** ^1^Institute of Agricultural Information and Economics, Shandong Academy of Agricultural Sciences, Jinan, China; ^2^School of Information Science and Engineering, Shandong Normal University, Jinan, China

**Keywords:** cascade decoder, cascade backbone network, Transformer, applet, disease classification

## Abstract

Fruit tree diseases are one of the major agricultural disasters in China. With the popularity of smartphones, there is a trend to use mobile devices to identify agricultural pests and diseases. In order to identify leaf diseases of apples more easily and efficiently, this paper proposes a cascade backbone network-based (CBNet) disease identification method to detect leaf diseases of apple trees in the field. The method first replaces traditional convolutional blocks with MobileViT-based convolutional blocks particularly for feature extraction. Compared with the traditional convolutional block, the MobileViT-based convolutional block is able to mine feature information in the image better. In order to refine the mined feature information, a feature refinement module is proposed in this paper. At the same time, this paper proposes a cascaded backbone network for effective fusion of features using a pyramidal cascaded multiplication operation. The results conducted on field datasets collected using mobile devices showed that the network proposed in this paper can achieve 96.76% accuracy and 96.71% F1-score. To the best of our knowledge, this paper is the first to introduce Transformer into apple leaf disease identification, and the results are promising.

## 1. Introduction

Fruits are indispensable in people's lives, and apples have long been known as the “king of fruits” because of their high yield, wide range of cultivation, and high survival rate. In addition, apples do not compete with grains and cotton for land. Therefore, it has very good economic benefits and is particularly suitable for large-scale planting. However, apple anthracnose leaf blight (ALB), apple leaf rust (ALR), apple leaf melasma (ALM), and apple leaf mosaic (AM) often cause harm to apples, and the spread, development speed is very fast, usually in a few days to a few weeks to spread to the whole leaf, the apple yield caused a great impact, which directly affects its economic benefits. Traditionally, the control of diseases in agricultural production is usually detected by farmers based on their own experience or expert's help, which is time-consuming and labor-intensive, and the vast majority of ordinary farmers do not receive guidance from experts in time. In this context, how to identify the apple diseases economically and effectively is an urgent question to research. In this article, we will address this problem with the help of the cascade backbone network with the mobile phone.

With the development of deep learning in recent years, more and more researchers have employed deep learning in the field of agriculture, and has made a great development in the research of plant disease identification. Deep learning techniques can automatically extract image features and classify plant disease spots, eliminating a lot of work such as feature extraction and manual design of classifiers in traditional image recognition techniques, with end-to-end features. Grinblat et al. (2016) firstly used deep learning methods to identify three kinds of legume: white bean, red bean, and soybean from leaf vein patterns *via* CNN. Ma J. et al. (2018) used deep convolutional neural networks to classify cucumber leaves collected in the field. Dandawate and Kokare (2015) proposed a convolutional neural network-based plant disease detection system using 800 cucumber leaves collected in a real production environment for four disease categories: anthracnose, downy mildew, powdery mildew and target leaf spot, and prevented overfitting by data enhancement, ultimately achieving an accuracy of 93.4%. K-fold cross-validation was utilized to prevent over-fitting, and an accuracy of 94.9% was finally achieved. Fuentes et al. (2017) proposed a deep learning-based approach to detect nine different tomato pests, combining VGG and ResNet networks as a “deep learning meta-architecture,” using cameras of different resolutions to capture images during data acquisition, and then proposing a local and global class annotation and data enhancement method to achieve an average mAP of 0.8306. Ferentinos (2018) used AlexNet, VGG networks, and GoogLeNet to classify plant diseases in the dataset. The dataset was trained using an open dataset (Hughes and Salathé, 2015) that contained 87,848 images of 25 different plants (including vigorous plants) from 58 different combinations of categories (plants, diseases) and ended up with an accuracy of 99.53%. Guo et al. (2022) used PlantScope images to extract spectral features commonly used in disease, pest, and vegetation growth monitoring as primary models, and used random forest and back propagation neural networks based on feature space optimization and the AdaBoost algorithm to construct a betel nut yellow leaf classification monitoring model with a Kappa coefficient of 0.765.

However, most deep learning-based methods are based on CNN networks for feature extraction. But too few layers of the network will lead to the inability to capture features at a distance and its performance will be limited by the perceptual field. While increasing the number of layers of the network will lead to problems such as higher computational overhead and increased resource consumption, and the pooling operation, which is often used together with CNN, will lead to the discarding of some of the location information, resulting in a loss of information, which can affect the effectiveness of the model to some extent. The commonly used attention mechanism is more dependent on external information, which often leads the network to focus on less important information.

In order to solve the problems caused by using only convolutional neural networks, this paper introduces Transformer. Transformer was first proposed by Vaswani et al. (2017), which completely discarded recursion and convolution and used only the self-attentive mechanism with good results. It was also widely used in NLP, such as Bert (Devlin et al., 2019), GPT (Radford et al., 2018, 2019; Brown et al., 2020) series, and ByT5 (Xue et al., 2021), etc. Dosovitskiy et al. (2020) first applied Transformer to the image domain and proposed the Vision Transformer model. It pioneered the Transformer in the field of vision, and many models were proposed on this basis, which greatly promoted the development of the field of computer vision. Mehta and Rastegari (2021) proposed MobileViT, which combined CNN and Vision Transformer, and used the advantages of these two models to successfully build a lightweight, low latency network for mobile vision tasks, lowering the threshold for using the Transformer. In this paper, the CNN module is partially replaced by a MobileViT-based convolutional block to extract information. A Feature Refinement (FR) module is proposed in order to uncover more connections between features. In order to better fuse the extracted features, we propose a cascaded backbone network that uses pyramidal concatenation to fuse the feature vectors.

The objectives of this study are (1) to extract features from images of apple leaves collected in the field and to build an apple leaf disease recognition module, (2) to validate the effectiveness of our proposed cascade decoder, Feature Refinement (FR) module, and Global Context-aware Block (GCB). The results of this study can provide a reference for the real-time classification of apple leaf diseases.

## 2. Materials and methods

### 2.1. Data collections

**Figure 1:**
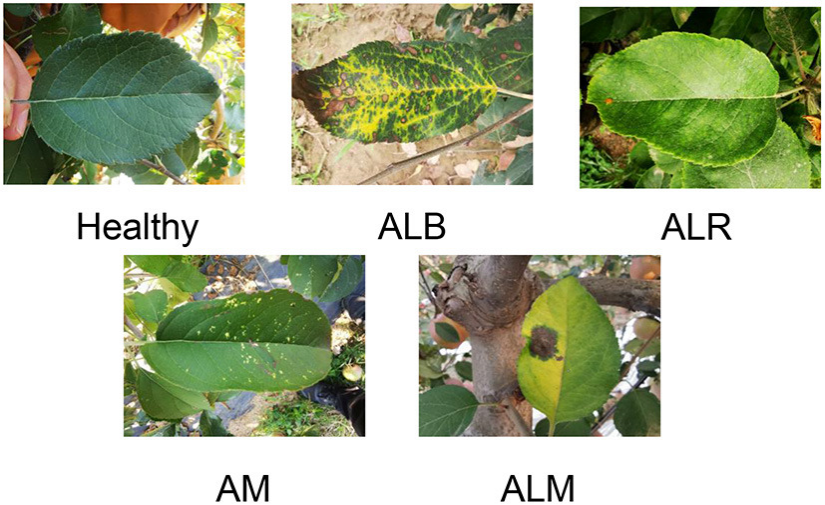
Status and classification of the dataset.

The main target of our study was leaf diseases of apples and the source of the dataset for our study was an orchard in Tai'an, Shandong Province, China. The total number of images is 765, as shown in Figure 1. After collection, we asked relevant experts to diagnose and annotate the apple leaf diseases. The processing time for collecting, diagnosing, and labeling each image was 3–5 min on average. By looking at the collected leaves and diagnosing them, we were able to identify four apple leaf diseases: Apple Anthracnose Leaf Blight (ALB), Apple Leaf Rust (ALR) disease, Apple Leaf Melasma (ALM), and Apple Mosaic (AM). In addition to ALB, ALR, ALM, and AM, images of healthy leaves were also collected. When selecting disease samples, we only considered the external visual characteristics of the disease.

ALB is a leaf blight of apples caused by the anthracnose fungus. When in a hot and humid environment, the symptoms are water loss in the form of scorched leaves, and the leaves will fall off eventually. When environmental conditions are not suitable, spots stop expanding and form dead spots in varying sizes on the leaves. Those with petiole onset, orange-yellow, slightly elevated, mostly fusiform, with small punctate sporangia on the initial surface and hair-like rust sporangia around the later spots, are ALR. ALM is usually manifested as the leaves are pin-awn radially expanding outward, the spots are small and many, the shape is not fixed, the spots have a lot of elevated small black spots, the later leaves gradually yellow. Similarly, leaves with a few yellow spots or bright yellow spots with clear margins were sampled for AM. Apple leaves appear larger blocks of dark green and light green discoloration, clear edges, number, and amount of small, this leaf is usually apple mosaic. Those lush leaves without any spots, were considered as healthy. See Figure 1 for pictures of healthy apple leaves and with diseases. Sixty-six pictures of healthy leaves, 435 pictures of ALB, 228 pictures of ALR, 70 pictures of ALM, and 80 pictures of AM were derived from these samples. We used a digital camera and a mobile phone to capture the apple leaves in the orchard, and to prevent other factors from affecting the quality of the acquired leaves, we used the default settings on the mobile phone.The focal length was 4 mm, exposure time was 1/60 s, ISO speed was 125, and the image size was 3,456 * 4,608. Unlike other work where the experimental dataset was obtained in a controlled environment indoors, our experimental dataset was obtained in a real production environment.

### 2.2. Data pre-processing

**Figure 2:**
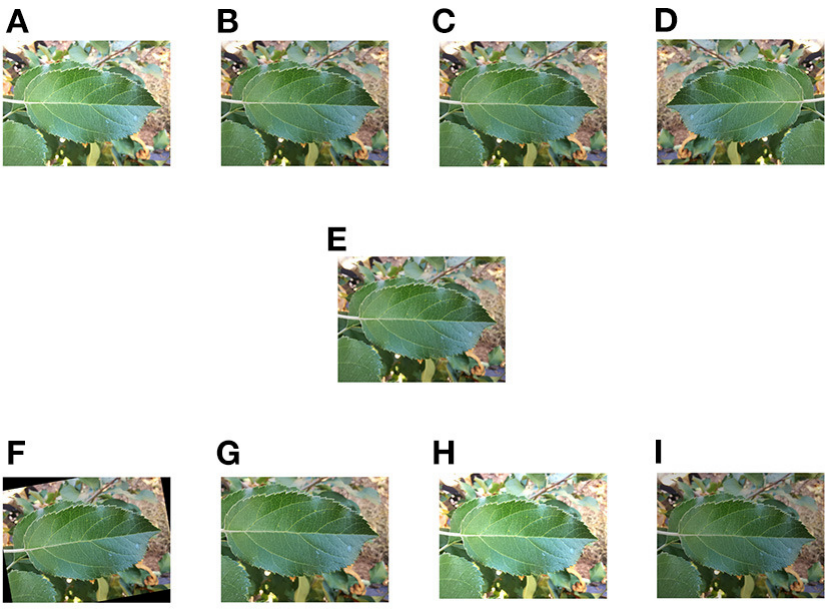
Schematic diagram of data enhancement. **(A)** Changing contrast. **(B)** Adding Gaussian noise. **(C)** Local zoom. **(D)** Mirroring operation. **(E)** Original image. **(F)** Random flip. **(G)** Random crop, **(H)** Increasing brightness. **(I)** Decreasing brightness.

Pre-processing includes normalization, image size setting and data enhancement. Normalization is usually done by compressing the image values to between 0 and 1 in order to speed up the convergence of the training network. The image size was set to a uniform 224*224. Data enhancement included adjusting the contrast, adding a Gaussian noise, image mirror flip operation, randomly flipping at an angle, random cropping and changes in brightness (as shown in Figure 2). Before data enhancement, we had a total of 459 training images and 153 tests and 153 validation images. The data enhancement was implemented in the pytorch (Paszke et al., 2019) framework, using the methods provided by OpenCV for enhancement. The collected dataset we divided the training set, test set and validation set in a ratio of 6:2:2.

### 2.3. Cascade backbone network

In order to make full use of the high level modal features of the RGB images and extract more semantic information, we divided the modal features of different levels into two groups: the high level feature group and the low level feature group, the low level feature group is F_1_= {Conv3 × 3, MobileNetV2, MobileViT}, the high level feature group is F_2_ = {MobileNetV2, MobileViT block, MobileNetV2}, with this grouping ensuring that the high level and low level features are retained, respectively.

**Figure 3:**
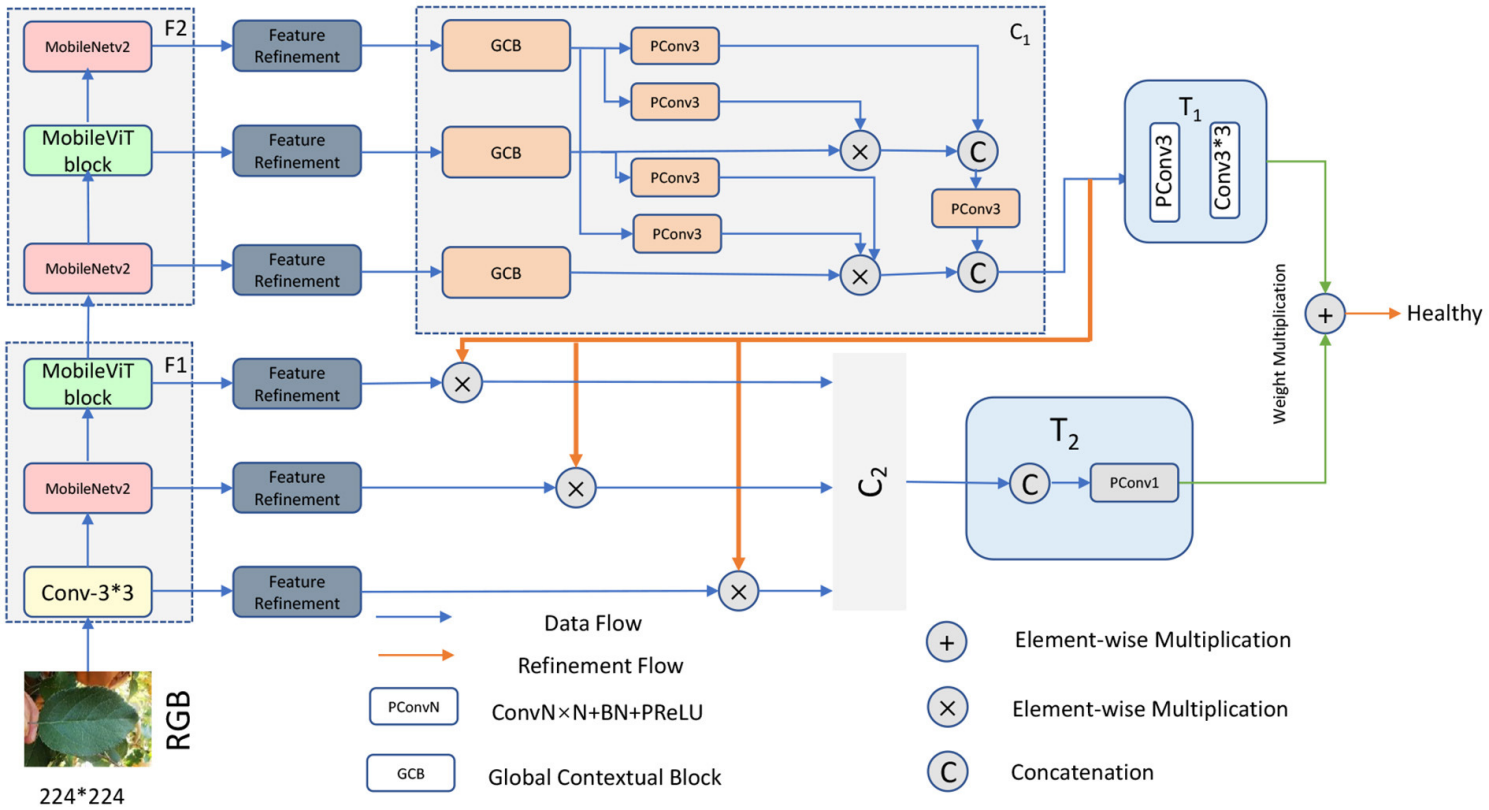
Architecture diagram of our proposed framework.

To make more efficient use of these two grouped features, our network uses a cascaded backbone network (Figure 3) which first generates a bootstrap feature (IF) using F_2_ and then uses this generated bootstrap feature to guide the learning process of F_1_. Using this network, our model is able to iteratively optimize the detailed information of the low-level features. This is because high-level features often contain rich global information, while low-level features carry a large amount of detailed information that facilitates the extraction of more feature information.

Specifically, we first extract feature information by MobileViT, and then the extracted feature information is refined by FR unit to obtain the refined modal features (${\text{F}}_{i}^{\text{r}}$, i=1,2...6), respectively. In the first stage, the three features,{${\text{F}}_{4}^{\text{r}}$,${\text{F}}_{5}^{\text{r}}$,${\text{F}}_{6}^{\text{r}}$} are aggregated by the first cascade decoder, a process that can be expressed as:


(1) $$\begin{array}{l} \text{IF}={\text{T}}_{1}\left({\text{C}}_{1}\left({\text{F}}_{4}^{\text{r}},{\text{F}}_{5}^{\text{r}},{\text{F}}_{6}^{\text{r}}\right)\right), \end{array}$$

Where IF represents the generated bootstrap features, C_1_ represents the first cascade decoder, and T_1_ represents the convolutional layer, which is used to reduce the number of channels. In the second stage, the bootstrap feature IF is used to guide the feature learning of the hybrid modality, a process that can be expressed as:


(2) $$\begin{array}{l} {\text{F}}_{\text{i}}{\text{r}}^{\text{′}}={\text{F}}_{\text{i}}\text{r}⊗\text{IF} \end{array}$$

Where ${\text{F}}_{\text{i}}^{\text{r}\prime }$(i=1,2,3) represents the optimized features, ⊗ represents the element multiplication operation, and then the three optimized features are aggregated by another cascade decoder, a process that can be expressed as:


(3) $$\begin{array}{l} \text{LC}={\text{T}}_{2}\left({\text{C}}_{2}\left({\text{F}}_{1}^{{\text{r}}^{\prime }},{\text{F}}_{2}^{{\text{r}}^{\prime }},{\text{F}}_{3}^{{\text{r}}^{\prime }}\right)\right) \end{array}$$

Where LC represents the learned features, T_2_ represents the Cat operation and the convolution layer, and C_2_ represents the second cascade decoder. We then process the features of these two with an element sum operation with additional weights, a process that can be expressed as:


(4) $$\begin{array}{l} \text{Final}=\alpha \times \text{IF}⊕\beta \times \text{LC,} \end{array}$$

Here Final denotes the final fused features, ⊕ represents element addition operations. The fused features are subjected to a fully connected (FC) layers and then subjected to a sigmoid function to output the predicted probabilities, a process that can be expressed as:


(5) $$\begin{array}{l} \text{Prediction}=\text{S}\left(\text{FC}\left(\text{Final}\right)\right) \end{array}$$

Here Prediction represents the type of prediction of the final output and S represents the Sigmoid activation function. Finally we supervise the process using the following loss function:


(6) $$\begin{array}{l} \text{L}=1_{\text{ce}}\left(\text{Prediction,}\text{ G}\right) \end{array}$$

Where, l_*ce*_ is the widely used binary cross-entropy loss function and G represents the true label. lce is calculated as following:


(7) $$\begin{array}{l} l_{ce}=\frac{1}{N}\underset{i}{\sum }L_{i}=-\frac{1}{N}\underset{i}{\sum }\overset{M}{\underset{c=1}{\sum }}y_{ic}\log\left(p_{ic}\right) \end{array}$$

Where *M* represents the number of categories, *N* represents the number of samples, y_*ic*_ represents the sign function (0 or 1), taking 1 if the category of sample i is equal to c and 0 otherwise, and p_*ic*_ represents the predicted probability that the observed sample i belongs to category c.

**Figure 4:**
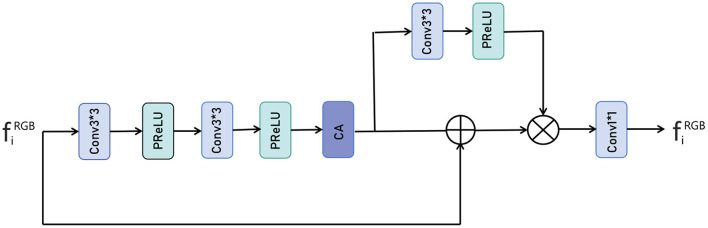
The proposed Feature Refinement (FR) module.

As shown in Figure 4, the input ${\text{f}}_{i}^{\text{RGB}}$ represents the side output features extracted from the RGB image, and we imported each side output feature to a series of weighting layers that consisted of two convolutional layers and two PReLU layers and a dimensional attention layer, a process that can be expressed as:


(8) $$\begin{array}{l} \text{Featur}{\text{e}}_{1}=\text{CA}\left(\text{Con}{\text{v}}_{3}\left(\text{PReLU}\left(\text{Con}{\text{v}}_{3}\left(\text{PReLU}\left({\text{f}}_{\text{i}}^{\text{RGB}}\right)\right)\right)\right)\right), \end{array}$$

(9) $$\begin{array}{l} \text{CA}={\mathrm{Conv}}_{3}\left(\text{PReLU}\left({\mathrm{Conv}}_{3}\left({\text{P}}_{\max}\left(\text{F}\right)\right)\right)\right)⊕\text{F}⊗\text{F}, \end{array}$$

Where Feature_1_ represents the extracted features, Conv3 represents the 3 × 3 convolution operation, PReLU represents the PReLU activation function, CA represents dimensional attention, *F* represents the input feature vector and ⊗ represents the element multiplication operation, ⊕ represents the element sum operation. After performing the above operations, we obtain more useful feature information and then use the unit of convolution plus PReLU to process the extracted feature information. This process can be expressed as following:


(10) $$\begin{array}{l} \text{Featur}{\text{e}}_{2}={\mathrm{Conv}}_{3}\left(\text{PReLU}\left({\text{Feature}}_{1}\right)\right), \end{array}$$

Where Feature2 represents the processed feature, and then the two parts are subjected to an elemental multiplication operation *via* a residual join, a process that can be expressed as:


(11) $$\begin{array}{l} \text{Featur}{\text{e}}_{3}={\text{Feature}}_{2}⊗\left({\text{f}}_{\text{i}}^{\text{RGB}}⊕{\text{Feature}}_{1}\right), \end{array}$$

Where, ⊗ represents the element multiplication operation, ⊕ represents the element sum operation, Feature3 represents the feature information after multiplication, and finally the number of fused feature channels is adjusted by 1 × 1 convolution. This process can be expressed as following:


(12) $$\begin{array}{l} \text{Fused}={\text{Conv}}_{1}({\text{Feature}}_{3})), \end{array}$$

Where Fused represents the final fused feature information and Conv1 represents the 1 × 1 convolution operation.

### 2.4. Cascade decoder

**Figure 5:**
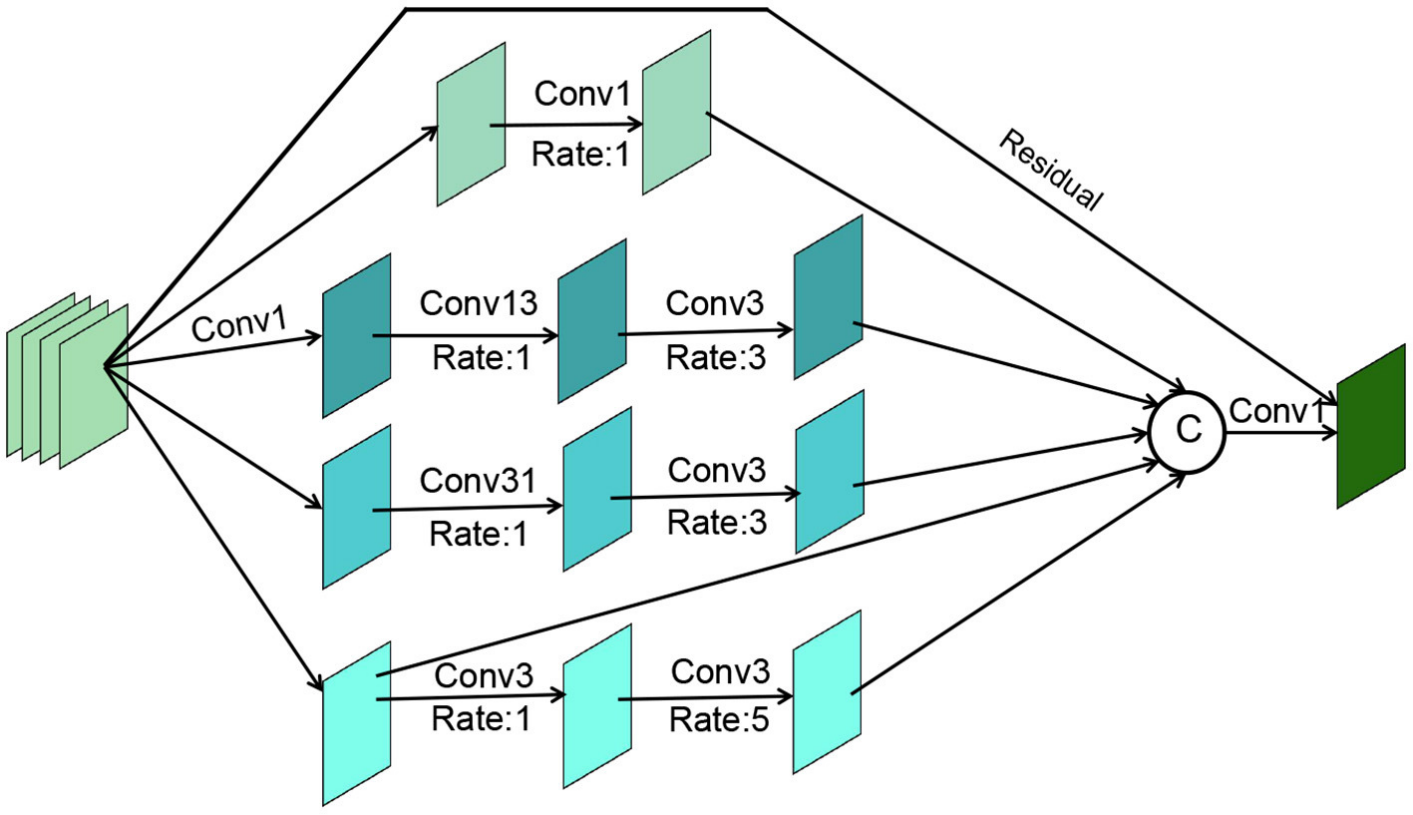
The proposed Global Context-aware Block (GCB) module.

For the above processed RGB features ${\text{f}}_{i}^{\text{RGB}}$(i ϵ {1,2,3...,6}) from different layers of the network, we need to efficiently exploit the multi-scale and multi-level information of the features within each group to help our cascade optimization strategy. As shown in Figure 5, our cascade decoder contains three GCB and a simple feature aggregation block. The GCB is an improvement on RFB-S (Ashqar and Abu-Naser, 2019), which contains a branch to expand the sense field and a residual connection to ensure that the original information is not lost. Specifically, the GCB module consists of four branches. The first step in the processing of all branches is to change their dimensionality using a 1 × 1 convolution operation. Then for the 2nd, 3rd, 4th, and 5th branches, a 1 × 1, 1 × 3, 3 × 1, and 3 × 3 convolution operation are performed respectively, all with an expansion rate of 1. Then for the 3rd, 4th and 5th branches, a 3 × 3 convolution operation is performed with an expansion rate of 3, 3, and 5. The aim here is to obtain more global information. Next, the four branches are stitched together and the number of channels is reduced by a 1 × 1 convolution layer. The final stitched features are concatenated with the residuals of the input features.

To further explore the associations between features, we use a pyramid-like multiplication and splicing operation for the features output from GCB. For each optimized feature, the parameters are updated by element-wise multiplication of features higher than it. Once the updates were completed, we subjected them to a splicing operation and then changed the number of channels by a 1 × 1 convolution operation. Finally, the feature vectors obtained in steps T_1_ and T_2_ were element-wise summed with additional weights. Here we took into account the different importance of the information contained in the features obtained from the high-level feature group F_2_ and the low-level feature group F_1_, for which we added a weight to each of the two different features, a process that can be expressed as follows:


(13) $$\begin{array}{l} \text{Final}=\gamma \times T_{1}+\delta \times T_{2} \end{array}$$

Where Final represents the features of T_1_ and T_2_ fusion, γ and δ represent their weights respectively. Final is then subjected to a full concatenation operation, followed by a sigmoid function,and finally the prediction type is output.

### 2.5. Applet for real-time leaf disease detection

**Figure 6:**
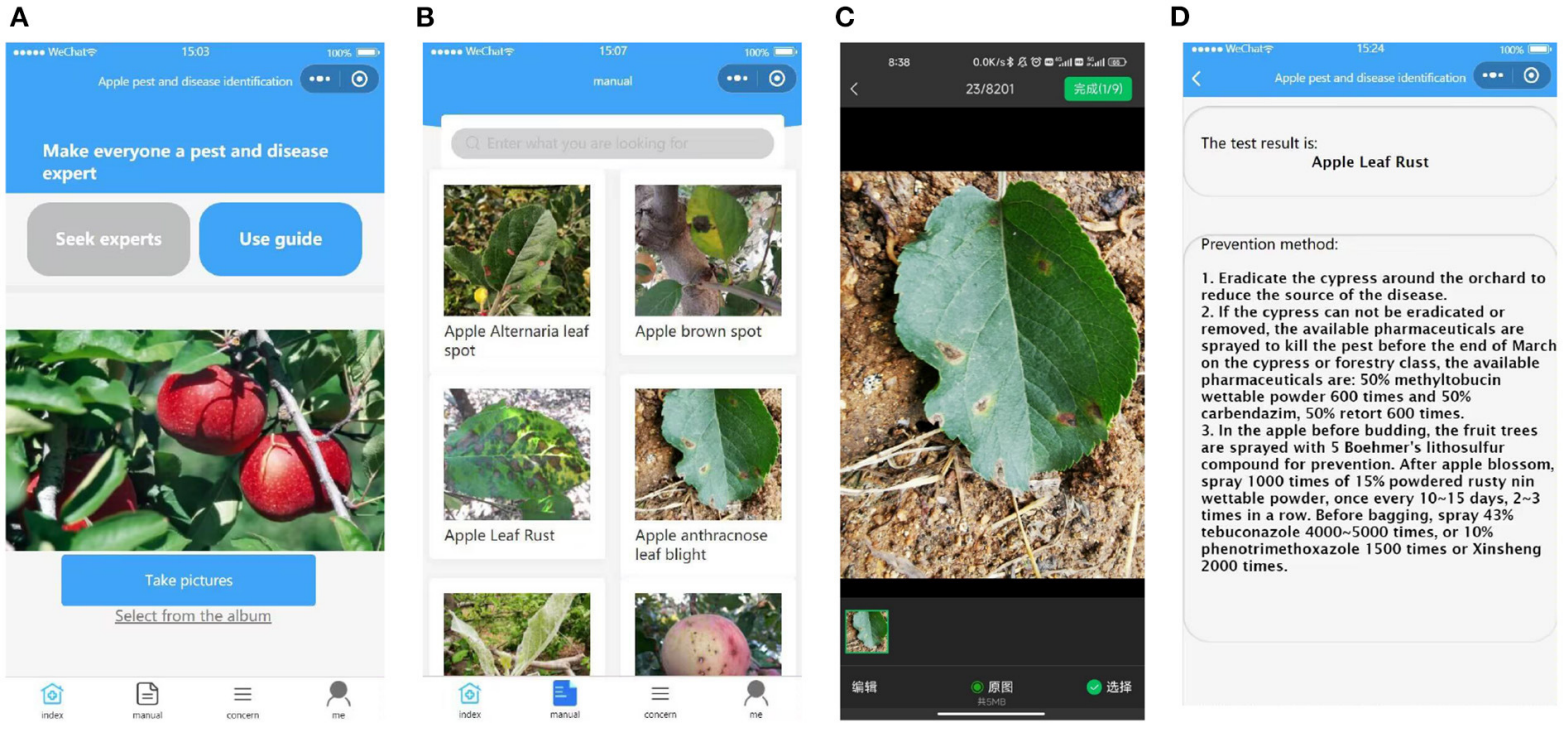
Introduction to the applet. From left to right, the main interface **(A)**, the catalog page **(B)**, the image selection page **(C)**, and the recognition results display and prevention suggestions **(D)**.

In order to detect apple disease leaves in real time, we developed a WeChat applet for use by fruit farmers to facilitate photo detection anytime and anywhere. In order to deploy the model of the algorithm to a smart phone, we stored the trained weight file as a .tar file, and then loaded this weight file and the front and back end of the program into the smart phone separately. The home page of the applet allows you to upload the photos that need to be identified, either by taking photos in real time or by selecting images from a folder (as shown in Figure 6A). The catalog page Figure 6B records pictures of different disease types, which can be displayed by clicking on them, making it easy for users to make preliminary comparisons. Select the image to be recognized in the selection page (Figure 6C), the results of the detection and the control method will be displayed after uploading Figure 6D.

## 3. Results and discussions

To evaluate the effectiveness of our proposed network, we compared several baseline networks in terms of different aspects. Our program implementation is based on PyTorch, using a Nvidia 2080Ti for training acceleration. To ensure the fairness of the experimental results, all comparison experiments were conducted according to the parameters in their text, using the same server and other parameter settings. All networks were trained with stochastic gradient descent (SGD), the learning rate was set to 0.1, and the weight decay was set to 4e-5 to mitigate overfitting problems. The maximum training generation for all models was 40, and the Dropout (Hinton et al., 2012) scale was set to 0.5.

### 3.1. Classification of the apple leaf dataset

**Figure 7:**
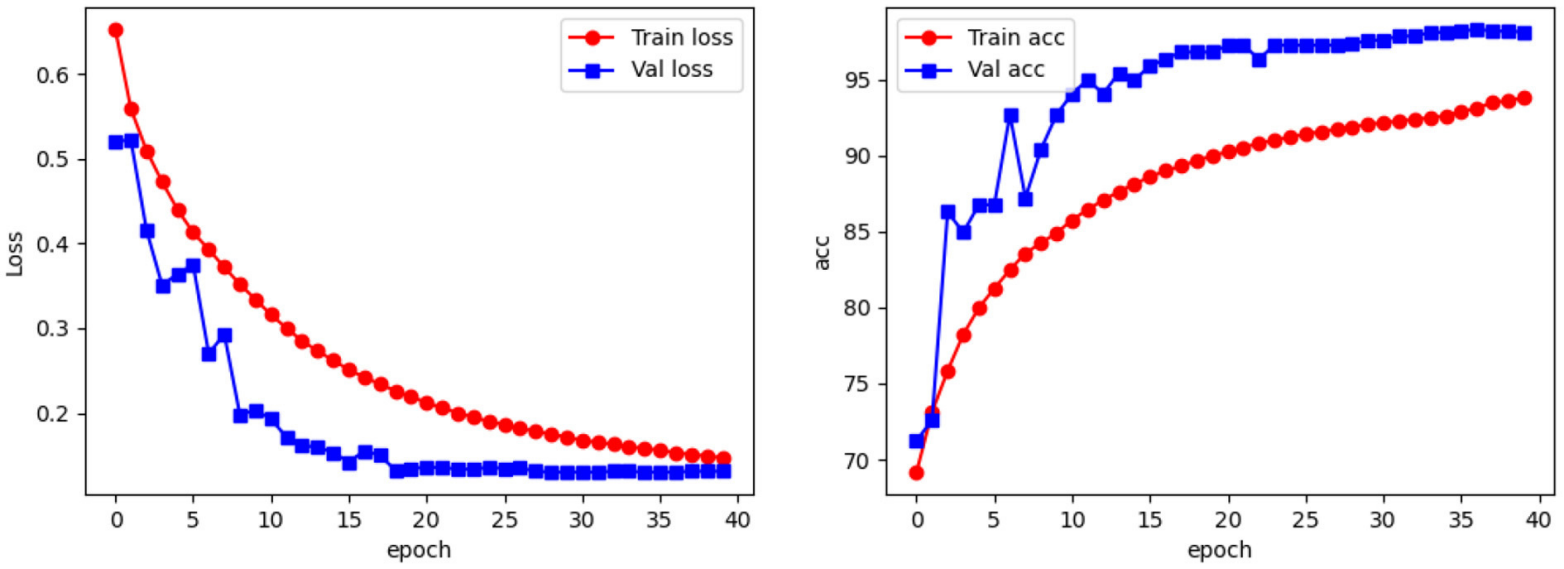
Accuracy of the network vs. loss function line graph.

To test the performance of our network in apple leaf classification, we used three metrics, accuracy, weighted F1-score and top-1, to measure the effectiveness of our models. Figure 7 shows a line graph of the loss and accuracy of our model during the training process. As can be seen, the model is slightly overfitted at the 3rd, 4th, 6th, and 7th epochs, but gradually returns to normal as the training progresses: from the 8th epoch onwards, the loss becomes smaller and tends to equalize, and the accuracy becomes larger and more stable. The best accuracy occurs at the 36th epoch, and then as the accuracy of the training set increases, the accuracy of the test set begins to decrease, at which point the model begins to overfit.

### 3.2. Comparison with other methods

#### 3.2.1. Baselines

We compare CBNet with the following baselines, which consists of three categories: basic CNN architecture, lightweight CNN architecture, and simple CNN architecture.

1). Basic CNN architecture. We chose the classic VGG-16 (Simonyan and Zisserman, 2015) model.

- **VGG16**: It uses several consecutive 3*3 convolutional kernels instead of larger ones, and the convolutional kernels all use the same convolutional kernel parameters. The model is composed of several convolutional and pooling layers stacked in such a way that it is easier to form a relatively deep structure.

2). Lightweight CNN architecture. We have selected several lightweight frameworks designed to simplify deep convolutional neural networks: MobileNet (Howard et al., 2017; Sandler et al., 2019) model and ShuffleNet (Ma N. et al., 2018; Zhang et al., 2018).

- **MobileNet**: MobileNet uses deeply separable convolution to significantly reduce the number of parameters and computation, allowing complex networks to be simplified into lightweight networks that can be deployed on mobile.
- **ShuffleNet**: To solve the drawbacks brought by group convolution, ShuffleNet proposes the method of using shuffle for different channels, which ensures the information exchange between the feature maps of different groups after group convolution.

3). Simple CNN architecture. We have chosen two simpler convolutional neural networks designed based on the characteristics of agricultural datasets with small sample sizes.

- **DCNN**:A deep convolutional neural network (DCNN, Ma J. et al., 2018) is proposed for symptom recognition of four cucumber diseases. Symptom images were segmented from cucumber leaf images collected under field conditions.
- **CNN**: Based on the characteristics of the image datasets they collect, several very simple methods (NIN-16, SENet-16, and WDenseNet, Xing et al., 2019) are proposed for the identification of citrus fruit diseases in terms of parameter efficiency.
- **CNN**: The authors trained a convolutional neural network (CNN, Ashqar and Abu-Naser, 2019) and collected a large number of tomato disease and health images under controlled conditions to achieve the identification of five tomato diseases using smartphone assisted disease surveillance.
- **SSCNN**: The authors collected smartphone-based citrus leaf disease dataset indoors, then designed a simple convolutional neural network SSCNN (Barman et al., 2020) based on the characteristics of the dataset and developed a cell phone software for real-time classification of citrus leaf disease.

#### 3.2.2. Results and analysis

**Figure 8:**
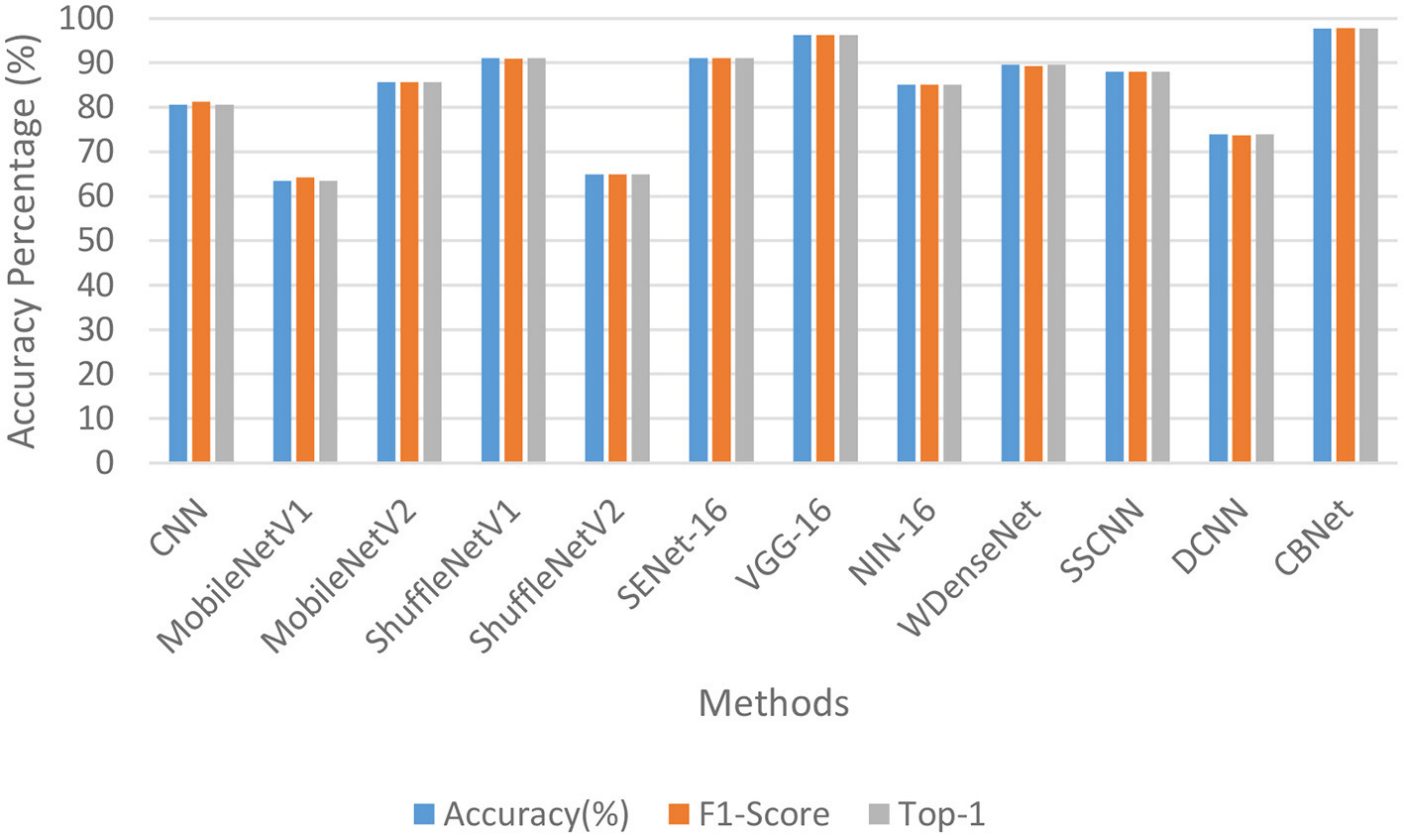
Histogram comparing CBNet with other methods.

Table 1 and Figure 8 show the quantitative metrics of our model and the other methods on the three quantitative measures, from which it can be seen that our model performs the best on all three metrics.

**Table 1:** Comparison of the classification results of our model and other models (bold represents the model with the best results).

**Models**	**Accuracy (*%*)**	**F1-score**	**Top-1**
CNN (Ashqar and Abu-Naser, 2019)	80.6	81.2	80.6
MobileNetV1 (Howard et al., 2017)	63.43	64.2	63.43
MobileNetV2 (Sandler et al., 2019)	85.64	85.62	85.64
ShuffleNetV1 (Zhang et al., 2018)	91.05	90.89	91.05
ShuffleNetV2 (Ma N. et al., 2018)	64.93	64.88	64.93
SENet-16 (Xing et al., 2019)	91.04	91.02	91.04
VGG-16 (Simonyan and Zisserman, 2015)	96.27	**96.54**	96.27
NIN-16 (Xing et al., 2019)	85.07	85.06	85.07
WDenseNet (Xing et al., 2019)	89.55	89.24	89.55
SSCNN (Barman et al., 2020)	88.06	87.98	88.06
DCNN (Ma J. et al., 2018)	73.88	73.75	73.88
CBNet	**96.76**	96.71	**96.76**

To demonstrate the advantages of our method applied on cell phones, we added two metrics, model parameters and average processing time per image [here we use Frames Per Second (FPS)]. As shown in Table 2, The basic rule of the number of parameters and the accuracy and inference time is that as the number of parameters increases, the accuracy of the model gradually increases and the inference time also increases accordingly. However, locally, some networks do not follow this pattern, such as MobileNetV1 and MobileNetV2, where the model inference speed becomes faster and the accuracy rate decreases as the number of parameters increases, which confirms that the classification ability of the model is not always positively correlated with the amount of model parameters. Because of the complex environment in apple orchards and the high timeliness of apple disease recognition, our program needs to maintain high accuracy and fast recognition time despite strong interference, and since the model is mounted on a cell phone, the model cannot be too large. Our model can achieve the best classification accuracy (over 95%), but the parameters are only 2.1% of VGG16, which is close to the number of parameters of MobileNet, but the classification effect is 14.2% higher than the better-performing MobileNetV2, and the inference speed is also comparable to MobileNet, which fully demonstrates the efficiency and lightness of our model.

**Table 2:** Comparison of the classification results of our model and other models (bold represents the model with the best results).

**Models**	**Params/M**	**FPS/ms**
CNN (Ashqar and Abu-Naser, 2019)	**2.6**	18.16
MobileNetV2 (Sandler et al., 2019)	8.5	13.55
MobileNetV1 (Howard et al., 2017)	12.25	10.6
ShuffleNetV1 (Zhang et al., 2018)	20.79	17.43
ShuffleNetV2 (Ma N. et al., 2018)	25.82	**6.78**
SENet-16 (Xing et al., 2019)	31.01	8.2
SSCNN (Barman et al., 2020)	34.2	14.1
DCNN (Ma J. et al., 2018)	34.46	33.59
WDenseNet (Xing et al., 2019)	62.7	15.08
NIN-16 (Xing et al., 2019)	68.32	16.68
VGG-16 (Simonyan and Zisserman, 2015)	512.21	61.09
CBNet	10.78	12.18

**Figure 9:**
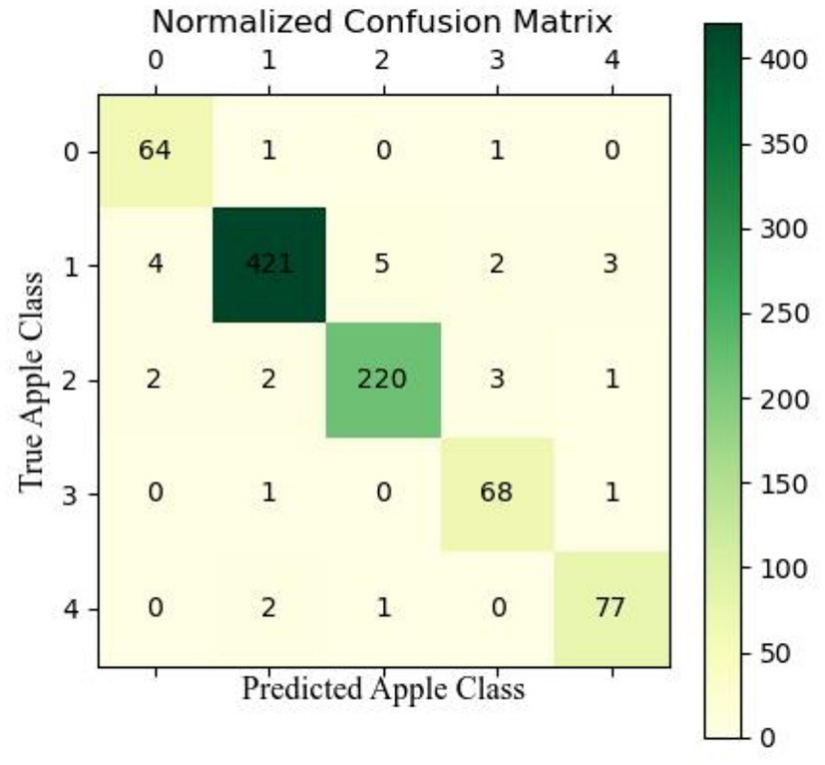
Confusion matrix diagram for CBNet.

We also used confusion matrix plots to evaluate our proposed model, as shown in Figure 9 (the types of diseases corresponding to the labels are shown in Figure 1). In the validation set, 64 images of healthy apple leaves were correctly classified, 2 were not correctly classified, 1 of which were misclassified as ALB and one was misclassified as AM. For an ALB, 421 images were correctly classified, but four was misidentified as a healthy apple leaf, while the other 10 were identified as ALR, AM and ALM. As for ALR, several apple diseases that have been almost misclassified as others. Next, ALR, 2 was misidentified as healthy, ALB or AM. Finally, for AM, a total of 77 were correctly classified, 2 were misidentified as ALB and ALR. As can be seen from the trials in the confusion matrix in Figure 9, ALB and ALR are often misclassified as each other's type, also because the two diseases have relatively similar appearance characteristics and they can be easily confused after the model extracts the features.

### 3.3. Ablation experiments

**Figure 10:**
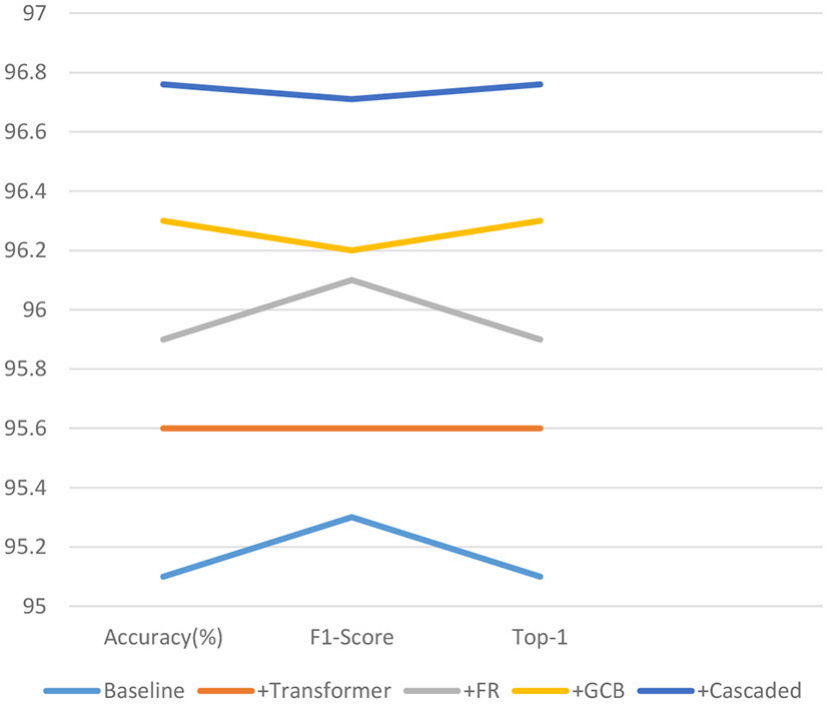
Line graph of the validity analysis of the proposed model modules on the three quantitative indicators.

As shown in Table 3 and Figure 10, the effectiveness of each module of our proposed model is analyzed. Where baseline refers to the standard convolution, pooling, and fully connected layer, i.e., using only convolution for feature extraction, followed by pooling to reduce redundant information, feature compression, and finally through a fully connected layer to output the predicted values. The +Transformer represents the replacement of the normal convolution operation of extracting feature values with our proposed feature extraction operation using MobileViT. +FR means that our proposed FR is added after feature extraction. +GCB means adding our proposed GCB module. +Cascaded means adding our proposed cascaded decoder module. As can be seen, each of these modules we propose improves the performance of our network to varying degrees, and the performance of our network on these three metrics confirms the effectiveness of the proposed modules.

**Table 3:** Comparison of the effectiveness of each module of CBNet (bold represents the model with the best results).

**Models**	**Accuracy (*%*)**	**F1-score**	**Top-1**
Baseline	95.1	95.3	95.1
+Transformer	95.6	95.6	95.6
+FR	95.9	96.1	95.9
+GCB	96.3	96.2	96.3
+Cascaded	**96.76**	**96.71**	**96.76**

## 4. Conclusions

In this paper, we propose a CBNet for a mobile collection device-based apple leaf disease classification system in the field, to the best of our knowledge, we are the first to use Transformer on the apple leaf disease classification task. To better mine the extracted features, we design a FR module that can extract more features from RGB images. To better combine the extracted features, we propose a cascade decoder and use a GCB module to more efficiently exploit the multi-scale and multi-level information within the feature set, and use a pyramidal concatenation operation for fusion between features to improve feature representability. Results indicate that our proposed network is very effective. This architecture is helpful for disease detection in apple leaves and provides new ideas for disease identification and classification of crops.

## Data availability statement

The original contributions presented in the study are included in the article/supplementary materials, further inquiries can be directed to the corresponding author/s.

## Author contributions

Conceptualization and methodology: XS, YZ, and CL. Software: YZ. Validation: XS and JZ. Investigation: XS, YZ, YF, and FW. Writing—original draft preparation: XS, FW, and CL. Writing—review and editing: FW, HR, YF, and JZ. Visualization: XS and YZ. Supervision: JZ, HR, and FW. Funding acquisition: JZ and FW. All authors have read and agreed to the published version of the manuscript. All authors contributed to the article and approved the submitted version.

## Funding

This research was funded by the Agricultural Scientific and Technological Innovation Project of Shandong Academy of Agricultural Sciences (Grant no. CXGC2022D07) and by the Collaborative Innovation Project between the Chinese Academy of Agricultural Sciences and Shandong Academy of Agricultural Sciences (Grant no. CAAS XTCX2018023).

## Conflict of interest

The authors declare that the research was conducted in the absence of any commercial or financial relationships that could be construed as a potential conflict of interest.

## Publisher's note

All claims expressed in this article are solely those of the authors and do not necessarily represent those of their affiliated organizations, or those of the publisher, the editors and the reviewers. Any product that may be evaluated in this article, or claim that may be made by its manufacturer, is not guaranteed or endorsed by the publisher.
